# Antiamoeba and Antitrypanosoma Activity of the Mexican *Plectranthus amboinicus* Essential Oil

**DOI:** 10.3390/pathogens15080801

**Published:** 2026-07-28

**Authors:** Juan Mauricio Ramírez-Vidal, Benjamín Nogueda-Torres, Rogelio Gómez-Escobedo, Laurence A. Marchat, Mariana Salas-Benito, Juan Santiago Salas-Benito, Ángel Ernesto Bañuelos-Hernández, Gildardo Rivera, Diana V. Navarrete-Carriola, Esther Ramírez-Moreno

**Affiliations:** 1 Laboratorio de Biomedicina Molecular 2, Escuela Nacional de Medicina y Homeopatía, Instituto Politécnico Nacional, Mexico City 07320, Mexico; jramirezv2000@alumno.ipn.mx (J.M.R.-V.); lmarchat@ipn.mx (L.A.M.); 2 Departamento de Parasitología, Escuela Nacional de Ciencias Biológicas, Instituto Politécnico Nacional, Mexico City 11340, Mexico; bnogueda@ipn.mx (B.N.-T.); rogelio.gomez.14@gmail.com (R.G.-E.); 3 Laboratorio de Biomedicina Molecular 3, Escuela Nacional de Medicina y Homeopatía, Instituto Politécnico Nacional, Mexico City 07320, Mexico; msalasb@ipn.mx (M.S.-B.); jsalas@ipn.mx (J.S.S.-B.); 4 Laboratorio de Enfermedades Osteoarticulares, Escuela Nacional de Medicina y Homeopatía, Instituto Politécnico Nacional, Mexico City 07320, Mexico; abanuelosh@ipn.mx; 5 Laboratorio de Biotecnología Farmacéutica, Centro de Biotecnología Genómica, Instituto Politécnico Nacional, Reynosa 88710, Mexico; giriveras@ipn.mx (G.R.); dnavarretec1900@ipn.mx (D.V.N.-C.)

**Keywords:** *Plectranthus amboinicus*, essential oil, *Entamoeba histolytica*, *Trypanosoma cruzi*

## Abstract

*Plectranthus amboinicus* (Loureiro) Sprengel is a plant whose essential oil has different properties; its composition varies according to the geographical area of cultivation. In this work, we aimed to identify by GC-MS the metabolites that are present in the essential oil of *P. amboinicus* grown in Mexico City and evaluate its effects against *Entamoeba histolytica* trophozoites and *Trypanosoma cruzi* trypomastigotes and epimastigotes. Additionally, its cytotoxicity on Vero cells was determined. Results showed that the essential oil of *P. amboinicus* cultivated in Mexico City is made up 46 compounds, belonging mainly to four classes of terpenoids, camphor being the major compound with an abundance of 30.64%, followed by alloaromadendrene oxide (14.43%), caryophyllene oxide (6.61%), aromadendrene oxide (6.47%) and carvacrol (5.8%). The essential oil possesses antiamoebic activity (IC_50_ of 121.7 μg/mL) and antitrypanosomic activity against trypomastigotes (LC_50_ = 61.66 μg/mL) and epimastigotes (IC_50_ of 210.8 μg/mL), being more effective against *T. cruzi* trypomastigotes, since it has an LC_50_ close to that of the reference drug benznidazole which is 25.72 µg/mL (98.83 µM). Future research will allow us to characterize the molecular effects that the essential oil of *P. amboinicus* cultivated in Mexico City has on the protozoan parasites *E. histolytica* and *T. cruzi*, and to compare the activity of the essential oil with respect to that of the individual metabolites, in mixtures and in combination with reference drugs.

## 1. Introduction

Plants are suppliers of immense resources for humans; from them it is possible to obtain food, spices, dyes, fibers, essences, wood and fuel. They are also a valuable source of new molecules with therapeutic potential for different diseases. It has been estimated that 80% of countries around the world use traditional medicine, including herbal medicine. The use of herbal medicines is high, increasing, and predominant in certain patient groups, cultures, and diseases. Additionally, more than 40% of pharmaceutical formulations are based on natural products [1].

*Plectranthus amboinicus* (Loureiro) Sprengel is a semi-succulent perennial plant whose genus has therapeutic, nutritional, horticultural, and pharmaceutical properties. It is one of the best-documented species in the Lamiaceae family and one of the most cited, especially for its medicinal properties, representing 68% of the usual applications of the genus [2]. As a medicinal plant, *P. amboinicus* is recognized for its usefulness in respiratory conditions, and infections caused by fungi, bacteria, and intestinal parasites, among many other uses, and it is currently the subject of various scientific studies for its promising medicinal properties [3].

Most of these properties have been observed in the essential oil of *P. amboinicus*, which has a variety of bioactive compounds. Notably, the essential oil obtained from the leaves and stem explants contains a total of 76 volatile constituents, and is particularly rich in phenolic monoterpenes. Its composition varies depending on the geographical area where the plant grew, the season of the year when it was collected and other variables such as the extraction method used, giving rise to different chemotypes, where thymol or carvacrol are frequently the major components [4,5,6]; however, other authors have reported 3-carene, 3-methyl-4-isopropylphenol or germacrene D as the main components of the essential oil of *P. amboinicus* [7,8,9].

The essential oil of *P. amboinicus* has various biological properties such as antioxidant, analgesic, anti-inflammatory, cytotoxic, antibacterial, antifungal, antiviral and larvicidal, among others [10]. Its activity has also been demonstrated against *Trypanosoma brucei* (IC_50_ = 34.9 µg/mL), *Leishmania amazonensis* (IC_50_ = 58.2 µg/mL), and *Plasmodium falciparum* (IC_50_ = 5.9 µg/mL), while it has no effect on amastigotes of *T. cruzi* and *L. infantum* [11]. Also, its antimalarial properties have been demonstrated in mice infected with *Plasmodium berguei* [12].

To the best of our knowledge, the effect of essential oil from *P. amboinicus* on the protozoan *Entamoeba histolytica* that causes human amoebiasis has not been evaluated, nor its effect against *T. cruzi* trypomastigotes and epimastigotes. Additionally, there is no information about the chemical composition of essential oil from *P. amboinicus* cultivated in Mexico, although it has been reported that it has antioxidant, antibacterial and enzyme-inhibiting properties [13]. Therefore, in this work, we aimed to determine the metabolites that are present in the essential oil of *P. amboinicus* grown in Mexico City and evaluate its effect on *E. histolytica* trophozoites and *T. cruzi* trypomastigotes and epimastigotes.

## 2. Materials and Methods

### 2.1. P. amboinicus Harvest and Essential Oil Extraction

*P. amboinicus* was cultivated and collected in Mexico City, Mexico, in August 2024; the plant was identified in the herbarium of the Facultad de Estudios Superiores Iztacala (FESI)-UNAM, Mexico by Edith Lopez Villafranco, and registered with voucher number 3039IZTA.

The amount corresponding to 200 g of leaves was placed in a ball flask with 600 mL of distilled water. Hydrodistillation was carried out at 150 °C for 3 h in a Clevenger apparatus. Three extractions of the essential oil were made, under the same conditions.

### 2.2. Essential Oil Characterization

The identification of the chemical components of the essential oil was carried out by GC-MS using the Agilent Technologies 7890B Network Series GC System (2850 Centerville Road Wilmington, DE 19808-1610 USA) and 5975C triple-axis mass selective detector, with an HP-5ms 19091S-433 capillary column (J & W Scientific, Folsom, CA, USA), 30 m × 250 μm × 0.25 μm.

For the chromatographic analysis, the following conditions were used: the injector temperature was 250 °C; the initial GC oven temperature was set at 40 °C for 5 min and then heated up to 140 °C at a rate of 5 °C/min and held at 140 °C for 5 min. Then the temperature was increased to 280 °C at a rate of 10 °C/min and held for 5 additional minutes with a final run time of 49 min. A volume of 2 μL (1 mg/μL) of the sample and standards was injected, with a column flow of 1.5 mL/min, a linear gas velocity of 33.61 cm/s, helium as the carrier gas, and source temperature of ions of 250 °C, start time of 3 min, end time of 49 min. The *m*/*z* detection occurred in a range of 50–550 *m*/*z*. Mass spectrometry was run in the electron impact mode (EI) at 70 eV. The chromatographic data were processed with the MS ChemStation and MS Interpreter programs. Mass spectra interpretation was confirmed by mass spectral library search using the National Institute Standards and Technology (NIST) and pure standards: carvacrol, thymol, camphor and caryophyllene oxide (Sigma Aldrich, St. Louis, MO, USA).

### 2.3. Evaluation of Antiamoebic Activity

Trophozoites of *E. histolytica* strain HM1-IMSS were grown at 37 °C in TYI-S-33 medium, supplemented with vitamins and adult bovine serum [14]. Trophozoites (7.5 × 10^4^) were treated for 48 h with 80–200 µg/mL of the essential oil of *P. amboinicus*, (dissolved in 0.02% DMSO and culture medium). The assays were done in triplicate and repeated three times. Trophozoites without treatment and treated with 0.02% DMSO or 9.5 µM metronidazole (Sigma-Aldrich Co., St. Louis, MO, USA) according to Bansal 2004 [15] were included as controls. The number of trophozoites was counted using a Neubauer chamber and the 50% growth inhibition concentration (IC_50_) was calculated with the GraphPad Prism program version 9.1.0.

### 2.4. Evaluation of Anti-Trypanosome Activity

The Mexican Ninoa strain of *T. cruzi,* obtained from a patient in the acute phase of Chagas disease [16], was used to evaluate the effect of *P. amboinicus* essential oil on the trypomastigote and epimastigote stages. The amounts of benznidazole to be used for trypomastigotes (98.83 µM) and epimastigotes (42.34 µM) of *T. cruzi* were determined, taking as a reference previously reported concentrations [17,18,19].

Blood trypomastigotes were obtained from *T. cruzi*-infected CD1 mice at the peak of parasitemia. Parasites (9 × 10^4^ per well) were seeded in a 96-well plate, treated with 3.12, 6.25, 12.5, 25, 50, and 100 μg/mL of *P. amboinicus* essential oil at 4 °C for 24 h. Each concentration was tested in triplicate, and the experiments were repeated three times. Trypomastigotes without treatment and treated with 0.02% DMSO or the reference drug benznidazole 98.83 µM (Sigma-Aldrich Co., St. Louis, MO, USA) were included as controls. Plates were incubated at 27 °C for 10 min to count the trypomastigotes. The 50% lysis concentration (LC_50_) was calculated using a concentration–response curve constructed using GraphPad Prism version 9.1.0.

*T. cruzi* epimastigotes were cultured in brain and heart infusion (BHI) medium supplemented with 10% fetal bovine serum (FBS), 100 U/mL of penicillin and 100 mg/mL of streptomycin at 27 °C. Epimastigotes (1 × 10^6^ in 90 µL) were seeded in a 96-well plate and treated with 3.12, 6.25, 12.5, 25, 50, 100, 200 and 400 µg/mL of *P. amboinicus* essential oil for 24 h at 27 °C. Epimastigotes treated with DMSO 0.02%, without treatment and treated with the reference drug benznidazole (42.34 µM) were included as controls. Then, 10 μL of MTT (5 mg/mL) were added to each well for 24 h, followed by 200 μL of SDS/HCl (10%/0.1%) for 24 h. Absorbance was read at 540 nm. The data were expressed as a percentage of viability; the concentration–response curve was constructed using the GraphPad Prism program version 9.1.0 to calculate the IC_50_.

### 2.5. Cytotoxicity Assays

Vero cells (derived from the kidney of the African green monkey, *Chlorocebus aethiops*) were cultured in supplemented DMEM medium (10% fetal bovine serum, glutamine, sodium bicarbonate, and penicillin-streptomycin) (Gibco) at 37 °C with 5% CO_2_. Cells were seeded in two 96-well plates (1 × 10^4^ cells/100 µL/well) and treated with 15.6, 31.25, 62.5, 125, 250, and 500 μg/mL of *P. amboinicus* essential oil. Cells without treatment and treated with 0.02% DMSO were included as controls. All experiments were done in triplicate. One plate was incubated for 24 h and the other for 48 h, which corresponds to incubation time used for each parasite. The culture medium was removed, 0.1 mL of 1.1 mM MTT was added, and the plates were incubated for 4 h at 37 °C and 5% CO_2._ After elimination of MTT, 90 µL of DMSO was added and plates were incubated for 15 min at 120 rpm agitation. Finally, the absorbance was measured at 570 nm. The 50% cytotoxic concentration (CC_50_) was calculated using the GraphPad Prism version 9.1.0 program.

The selectivity index (SI) of the essential oil on *E. histolytica* and *T. cruzi* was calculated using the CC_50_ obtained in Vero Cells at 48 h and 24 h, respectively, with IC_50_ and LC_50_ obtained for each parasite.

### 2.6. Statistical Analysis

Data were compared using the one-way ANOVA statistical test and a multiple comparison post hoc test with the Dunnett method, using Microsoft Excel and the Real Statistics add (RealStats 2024) on for the same software. *p* values < 0.05 were considered as statistically significant.

## 3. Results

### 3.1. The Major Component of the Essential Oil of P. amboinicus Is Camphor

**Figure 1:**
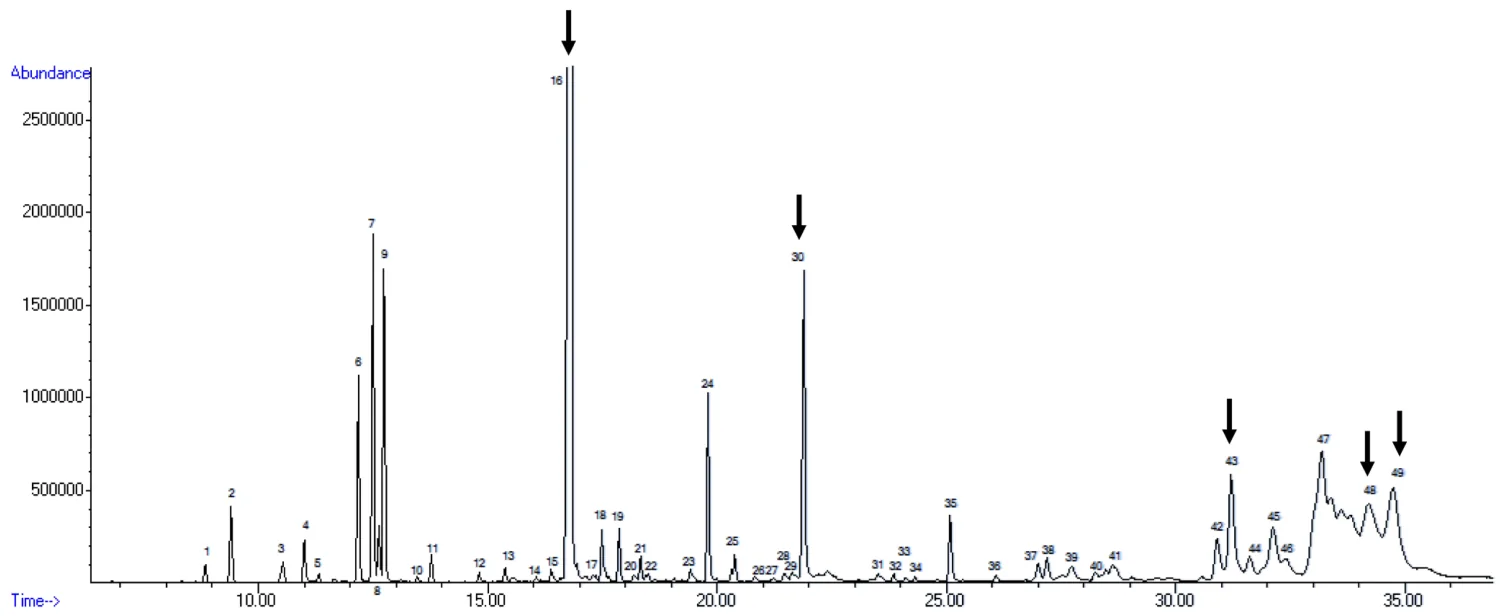
Representative chromatogram of the essential oil of *P. amboinicus* obtained by gas chromatography. The numbers at each peak correspond to the compounds listed in Table 1. The arrows point to the most abundant molecules.

We obtained between 250 and 300 µL of *P. amboinicus* essential oil from 200 g of leaves, using the hydrodistillation method. The components of the essential oil of *P. amboinicus* grown in Mexico were determined by gas chromatography coupled with mass spectrometry. The chromatographic data (Figure 1) were processed with the MS ChemStation and MS Interpreter program; the identified compounds are shown in Table 1. The essential oil is made up of 49 compounds, belonging mainly to four classes of terpenoids: Monoterpene hydrocarbon (11 molecules), oxygenate monoterpene (20 molecules), sesquiterpene hydrocarbon (eight molecules) and oxygenated sesquiterpenes (eight molecules). The five most abundant molecules in the three extractions were Camphor (30.64%, peak 16), Alloaromadendrene oxide (14.43%, peak 48), Caryophyllene oxide (6.61%, peak 43), Aromadendrene oxide (6.47%, peak 49) and Carvacrol (5.8%, peak 30), with slight variations in their abundance among the three extractions.

### 3.2. The Essential Oil of P. amboinicus Inhibits E. histolytica Trophozoites Growth

**Figure 2:**
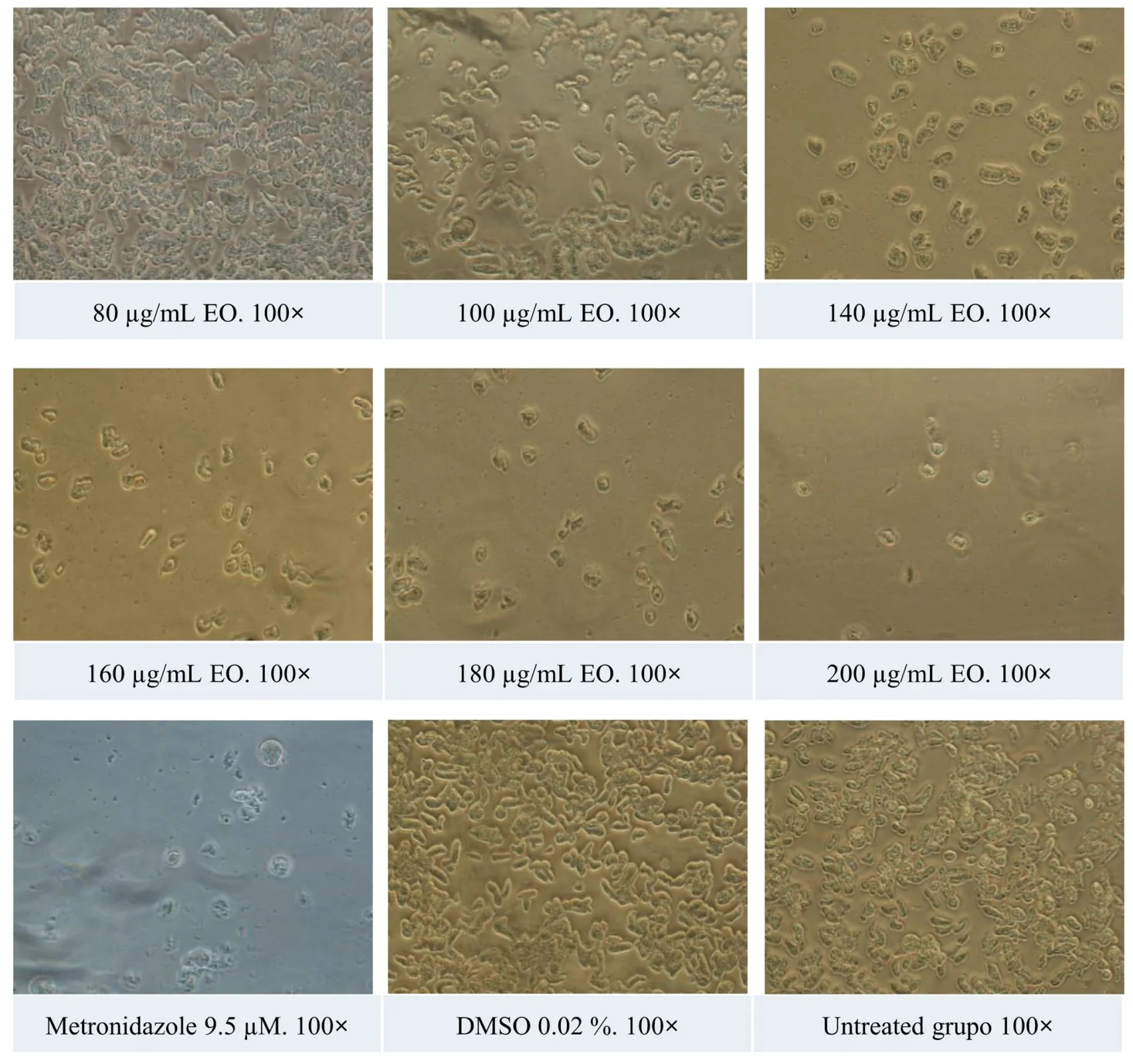
*E. histolytica* trophozoites treated with increasing concentrations of *P. amboinicus* essential oil (EO)**.** The trophozoites were treated for 48 h with the essential oil. Controls without treatment and treated with DMSO and metronidazole were included.

To evaluate the effect of essential oil of *P. amboinicus on E. histolytica* trophozoites, parasites were treated with increasing concentrations of essential oil for 48 h. Results showed that untreated and vehicle (DMSO)-treated cells showed no change and maintained a uniform monolayer. Likewise, treatments with up to 80 µg/mL of the essential oil had no apparent effect on trophozoites, which grow in a uniform monolayer, exhibiting the classical amoeboid morphology with pseudopods emerging from its periphery. On the other hand, from 100 µg/mL, a reduction in the number of parasites was observed with a dose-dependent effect, reaching a scarce confluence with 200 µg/mL (<10% approximately). Additionally, some morphological changes occurred, such as the presence of some rounded cells, and a decrease in cell size from 160 µg/mL of essential oil. The trophozoites treated with metronidazole (9.5 µM) presented characteristics comparable to those treated with 200 µg/mL of essential oil (Figure 2).

**Figure 3:**
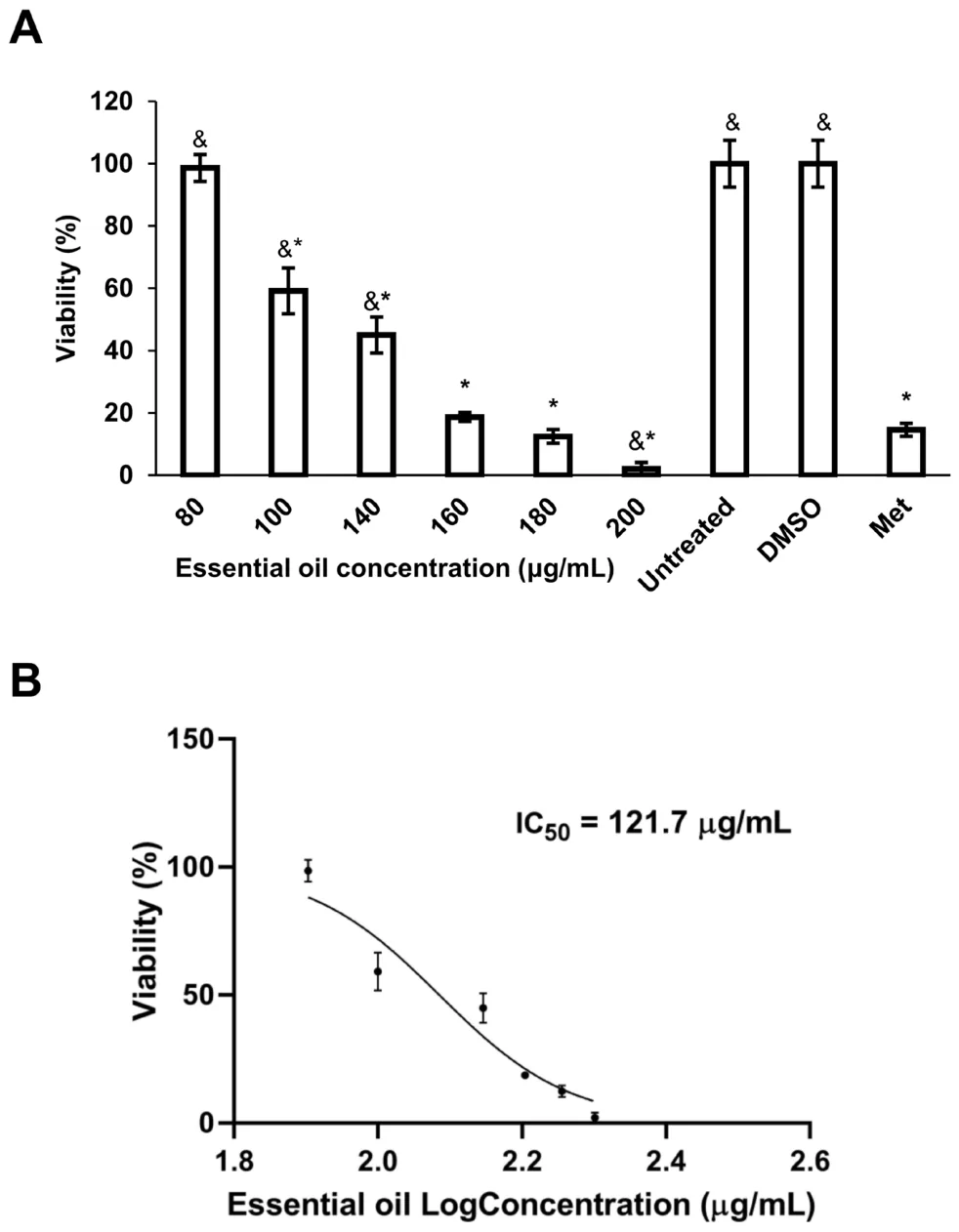
Effect of the essential oil of *P. amboinicus* on the viability of *E. histolytica*. Trophozoites were treated for 48 h with different concentrations of the essential oil of *P. amboinicus*. (**A**) Graph of the viability of *E. histolytica* trophozoites with respect to the concentration of essential oil. Data were analyzed with an ANOVA test; subsequently, a Dunnett’s test was performed. * *p* < 0.05 vs. untreated cells; & *p* < 0.05 vs. metronidazole (Met)-treated cells. (**B**) Concentration–response curve of the effect of the essential oil of *P. amboinicus* on trophozoites of *E. histolytica*. The IC_50_ value obtained by this method is 121.7 ± 8.3 μg/mL with a 95% confidence interval. Bars represent the standard deviation. *n* = 3.

The number of trophozoites was determined and viability was expressed with respect to the group without treatment. The results shown in Figure 3A evidenced that treatment with DMSO and up to 80 µg/mL of *P. amboinicus* essential oil did not affect parasite proliferation, while parasite number was reduced in a concentration-dependent way from 100 µg/mL. Additionally, the use of *P. amboinicus* essential oil at 160 µg/mL and 180 µg/mL produced a similar inhibitory effect to metronidazole (9.5 µM), while the highest concentration of 200 µg/mL was more effective than metronidazole and almost abolished cell viability. Using a non-linear regression, the IC_50_ of 121.7 ± 8.3 µg/mL was calculated (Figure 3B).

### 3.3. The Essential Oil of P. amboinicus Decreases Viability of T. cruzi Trypomastigotes and Epimastigotes

**Figure 4:**
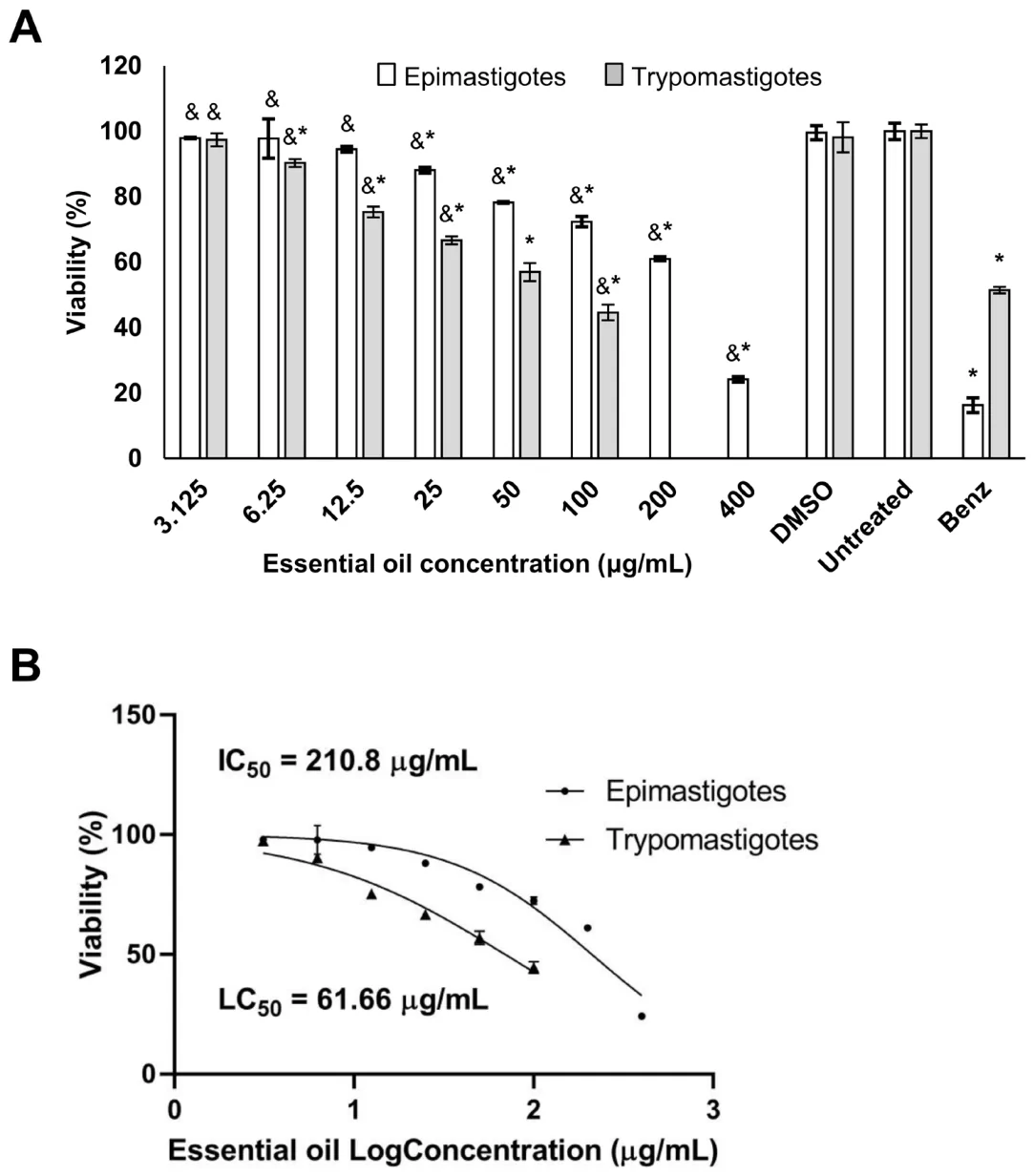
Effect of the essential oil of *P. amboinicus* on the viability of *T. cruzi*. Parasites were incubated for 24 h with different concentrations of the essential oil and viability was determined. (**A**) Graph of the viability of *T. cruzi* epimastigotes (white columns) and trypomastigotes (gray columns), with respect to the concentration of essential oil. Bars represent the average of three independent experiments. Data were analyzed with an ANOVA test and Dunnett’s test. * *p* < 0.05 vs. untreated cells; & *p* < 0.05 vs. benznidazole (Benz)-treated cells. (**B**) Concentration–response curve of the effect of the essential oil of *P. amboinicus* on *T. cruzi* epimastigotes and trypomastigotes. The IC_50_ and LC_50_ are shown, with a 95% confidence interval. n = 3.

To evaluate whether the essential oil of *P. amboinicus* affects *T. cruzi,* trypomastigotes were incubated for 24 h with increasing concentrations of the essential oil. Cells were counted and the LC_50_ was determined. As shown in Figure 4A (gray columns), concentrations of 6.25, 12.5, 25, 50 and 100 µg/mL significantly reduced trypomastigotes viability between 10 and 56% with respect to the control group without treatment, while the lowest concentration (3.125 µg/mL) had no significant effect. Similarly, DMSO did not affect trypomastigotes viability. Additionally, the use of essential oil at 50 µg/mL of *P. amboinicus* essential oil produced a similar inhibitory effect to benznidazole (98.83 µM), while the highest concentration of 100 µg/mL was more effective than benznidazole. Using a non-linear regression from the concentration–response curve, an LC_50_ of 61.66 ± 8.42 μg/mL was obtained (Figure 4B).

In another assay, increasing concentrations of essential oil were tested on *T. cruzi* epimastigotes for 24 h. Concentrations of 25, 50, 100, 200, and 400 µg/mL reduced the viability of epimastigotes between 12 and 76%, while treatments with 3.125, 6.25 and 12.5 µg/mL did not exhibit significant differences when compared with control untreated cells (Figure 4A, white columns). Additionally, the use of essential oil at 400 µg/mL produced a similar inhibitory effect to benznidazole (42.34 µM). An IC_50_ of 210.8 ± 28.2 μg/mL can be determined from the concentration–response curve (Figure 4B).

### 3.4. Cytotoxicity of P. amboinicus Essential Oil in Mammalian Cells

**Figure 5:**
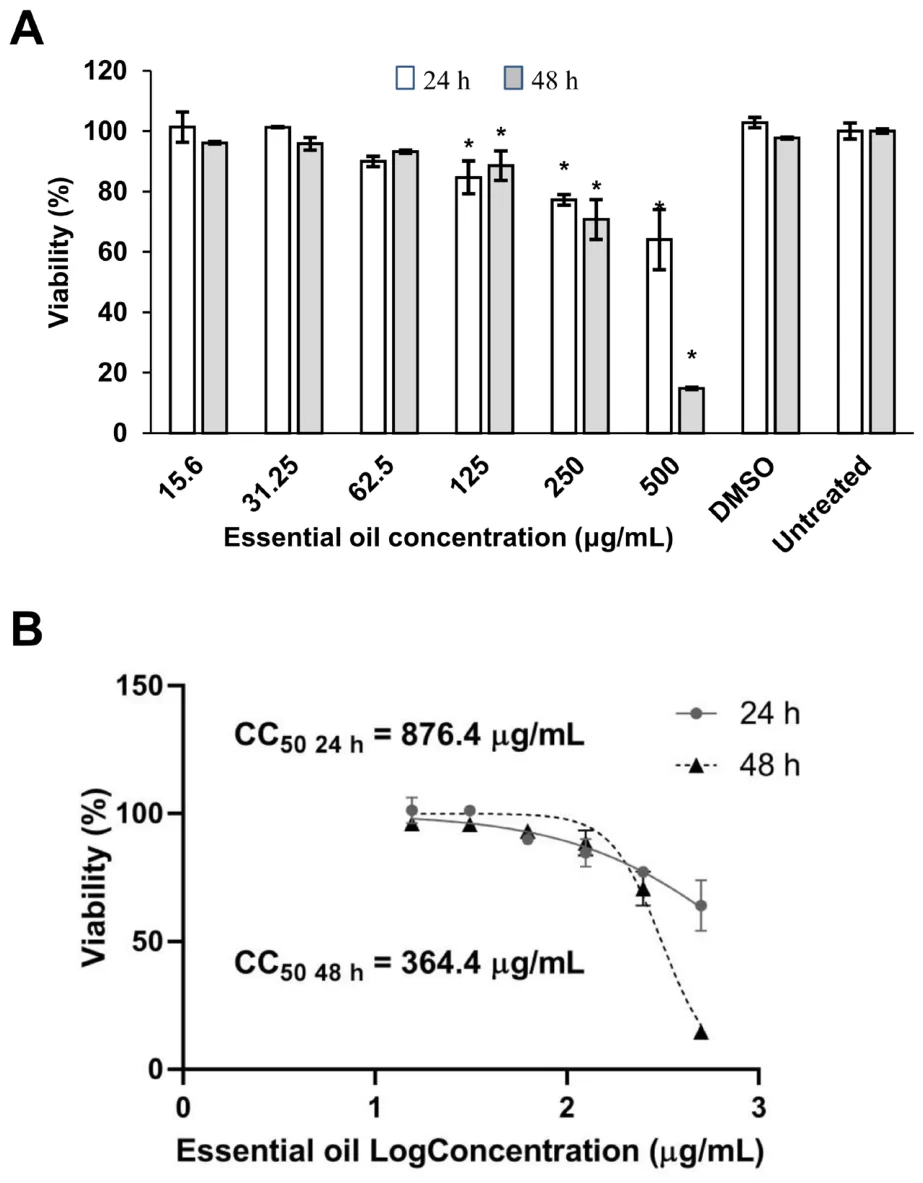
Effect of the essential oil of *P. amboinicus* on the viability of Vero cells at 24 and 48 h. A total of 1 × 10^4^ cells were incubated for 24 and 48 h with different concentrations of the essential oil. Viability was determined using the MTT technique. (**A**) Graph of the relationship of the concentration of essential oil in reference to the viability of Vero cells at 24 h (white columns) and 48 h (gray columns). Data were analyzed with an ANOVA test and a Dunnett test, * *p* < 0.05 vs. untreated cells. Bars represent the standard deviation. *n* = 3. (**B**) Concentration–response curve of the effect of the essential oil of *P. amboinicus* on Vero cells at 24 and 48 h. The CC_50_ obtained is 876.4 ± 233.6 μg/mL at 24 h and 364.4 ± 113.2 μg/mL at 48 h with a 95% confidence interval. *n* = 3.

The toxicity of the essential oil of *P. amboinicus* on mammalian cells (Vero cells) was assessed using the MTT assay at 24 and 48 h, which correspond to incubation time used for each parasite. The essential oil at concentrations of 15.6, 31.25, 62.5 and 125 µg/mL did not produce a decrease in cell viability greater than 80%, even if the concentration of 125 µg/mL showed a significant difference with respect to cells without treatment. Concentrations of 250 and 500 µg/mL of essential oil produced a decrease in viability greater than 80%. DMSO had no significant effect on Vero cell viability (Figure 5A). From the concentration–response curve corresponding to 24 h, a CC_50_ of 876.4 ± 233.6 μg/mL was obtained, while the CC_50_ value was 364.4 ± 113.2 µg/mL at 48 h (Figure 5B).

### 3.5. The Essential Oil of P. amboinicus Is More Selective Against T. cruzi Trypomastigotes

The selectivity index of the essential oil of *P. amboinicus* was calculated from the values of IC_50_, LC_50_ and CC_50_ described above, revealing that it is more selective against *T. cruzi* with an SI of 14.21 and 4.16 for trypomastigotes and epimastigotes, respectively. The SI for *E. histolytica* was 2.99 (Table 2).

## 4. Discussion

*P. amboinicus* (Loureiro) Sprengel is a plant with invaluable biological properties attributed to the composition of its essential oil, which varies depending on many factors, including the geographical region where it is cultivated. In this work, we identified by GC-MS the metabolites present in the essential oil of *P. amboinicus* cultivated in Mexico City and evaluated their effects against trophozoites of *E. histolytica* and trypomastigotes and epimastigotes of *T. cruzi*, as well as their cytotoxicity in Vero cells.

Thymol or carvacrol are frequently identified as the major components of the essential oil of *P. amboinicus* [4,5,6]; however, we found that the major component is camphor with an abundance of 30.64%, followed by alloaromadendrene oxide, caryophyllene oxide, aromadendrene oxide and carvacrol. All these compounds have been previously reported as components of the essential oil of this plant, and some of them are in considerable abundance [4,20,21,22,23,24].

Like us, other authors have found other major compounds, for example, Uma et al. [8] reported that the predominant compounds are 3-methyl-4-isopropyl phenol (31.70%), squalene (10.07%), phytol (8.44%), durohydroquinone (2.73%) and caryophyllene (2.36%) using plants collected in India; Erny et al. [7] identified 3-carene as the most abundant component (20.78%), followed by carvacrol (19.29%) and camphor (17.96%) in *P. amboinicus* harvested in Malaysia; while Aguiar et al. [9] reported germacrene-D as the most abundant compound (38.6%), followed by (E)-caryophyllene (18.91%) and copaene (8.03%), in the essential oil of Brazilian plants. Mexico City is located at an altitude of 2240 m above sea level (latitude 19°15′42″, longitude 99°07′39″), and at the time of plant collection, a warm climate was reported, with an average temperature of 23 °C [25]. The specific chemical composition of the essential oil of *P. amboinicus* cultivated in Mexico City is probably due to its geographical location, as well as local environmental and seasonal factors, as has been reported for the composition of the oil collected in other countries [4,26]. Therefore, its biological effect could be different from that described for oils from *P. amboinicus* grown in other parts of the world.

Most of the compounds identified in the oil are terpenoids, which have been shown to have various biological effects, particularly antiprotozoal properties. Camphor (13.0%) and caryophyllene oxide (10.87%) are the main components of the essential oil of *Artemisia indica*, which exhibits in vitro effect against *P. falciparum*, *T. brucei rhodesiense*, *T. cruzi* and *L. donovani,* as well as a potential malaria prophylactic effect by inhibiting at least two recombinant plasmodial fatty acid biosynthesis (PfFAS-II) enzymes [27]. Similarly, the dichloromethane extract of the aerial parts of *Artemisia abyssinica* has high activity against bloodstream forms of *T. brucei*, and camphor was found to be the principal compound (38.73%) of the extract [28]. On the other hand, camphor has also shown antifungal properties against different candida species, reducing fungal virulence traits such as biofilm establishment and hyphae formation, as well as deregulating the expression of CDR1 and CDR2 genes coding for fungal efflux pumps [29]. Additionally, transcriptomic analysis showed that camphor deregulates the ECE1, ECE2, RBT1 and EED1 genes, which are specific to hyphae and related to biofilm formation [30]. In addition, camphor has strong activity against four species of *Fusarium*, producing absolute inhibition of the growth of phytopathogenic fungi, while seriously interfering with cell wall synthesis, suppressing mycelium growth and increasing cell permeability [31]. The antibacterial properties of camphor-impregnated nanogel have been demonstrated in vitro against *Pseudomonas aeruginosa*, *Staphylococcus aureus*, *Listeria monocytogenes*, and *Escherichia coli* [32].

There is little information on the biological activity of the major aromadendrene-type sesquiterpenes identified in the essential oil of *P. amboinicus* grown in Mexico. Previous studies show that alloaromadendrene and aromandendrene affect the blood stream form of *T. brucei* with an IC_50_ of 1.9 µg/mL and 18.2 µg/mL, respectively, while they showed poor activity on promastigotes of *Leishmania major* [33]. On the other hand, molecular docking studies indicated that the interaction of alloaromadendrene oxide with the glucosyl transferase protein interferes with its binding to sucrose in *Streptococcus mutans* [34]; additionally, alloaromadrendrene oxide has high binding affinity for the ATP-binging pocket of the bacterial gyrase B enzyme [35]. Regarding the effect of aromadendrene oxide in other cell types, it has been shown to inhibit the growth of human squamous cell carcinoma and precancerous cells by arresting the cell cycle and the mitochondrial apoptosis pathway [36]. Furthermore, aromadendrene oxide has biopesticidal activity against larvae of insects considered forest pests (*Hyblaea puera*, *Eligma narcissus*, and *Atteva fabriciella*), causing 65–70% mortality within 72 h [37].

With respect to caryophyllene oxide, its effect on trypanosomatids has already been documented. In *L. tarentolae*, it affects the electron transport chain, strongly inhibiting oxygen consumption and complex III of the mitochondrion [38]. Mixed with lupenone, caryophyllene oxide (in a 1:4 ratio) exerts a synergistic effect against *T. cruzi* epimastigotes in vitro and reduces the number of amastigotes by more than 80% in cardiac tissue and skeletal muscle of infected mice; the 1:1 lupenone-caryophyllene oxide ratio had a smaller effect (IC_50_ = 80.3 µg/mL), while ratios of 2:3, 3:2, and 4:1 indicated an antagonistic effect, suggesting that the mixture of both terpenoids in a 1:4 ratio is necessary for its effective trypanocidal activity and that high proportions of lupenone result in low activity [39]. Similarly, caryophyllene oxide with limonene showed a better pharmacological interaction across all three parasite stages, with the advantage of reduction in the individual cytotoxicity of each terpene in Vero cells. Interestingly, limonene was also found to show a strong synergistic interaction with benznidazole, reducing its IC_50_ by 17 times, and carvone, the compound with the lowest trypanocidal activity, showed a possible antagonistic effect with limonene, likely due to its high antioxidant capacity [40].

Regarding carvacrol, it exhibited a significant in vitro activity against trypomastigotes of *T. brucei rhodesiense,* and a moderate activity against amastigotes of *L. donovani* and the blood-stage forms of multidrug-resistant *P. falciparum* parasites, while it showed low activity against *T. cruzi* trypomastigotes [41]. Additionally, carvacrol inhibited *E. histolytica* trophozoites by 95–98% [42]. In bacteria, carvacrol alters the structure and function of the cytoplasmic membrane of *Listeria monocytogenes*, modifying its fluidity and its permeability for H^+^ and K^+^ ions, producing changes in the membrane potential and leading to cell death; additionally, it produces cell wall and membrane destruction, and disruption of nucleotide metabolism and respiratory activity [43,44].

Another example of synergism among the components of an essential oil was reported in the essential oil of *Origamun vulgare*, containing carvacrol, thymol, γ-terpinene, p-cymene, and β-caryophyllene as major components. This study found that the essential oil has an IC_50_ of 16 µg/mL against *L. amazonensis* promastigotes, which is better than that obtained by testing carvacrol or thymol alone. This also demonstrates that the combination of compounds is important for the biological effect and suggests that the minor components are critical for synergistic activity [45].

Altogether, these data suggest that the main components of the essential oil of *P. amboinicus* grown in Mexico, may have the potential to affect *E. histolytica* and *T. cruzi* viability. Congruently, our results evidenced for the first time the antiamoebic effect of this essential oil with an IC_50_ of 121.7 µg ± 8.3 µg/mL, which is in the range considered weak or moderate by other authors who evaluated the effect of plant extracts and other essential oils [46,47,48]. On the other hand, the SI obtained was low (2.99), which could indicate a potentially toxic effect of the oil at that concentration for its use as an antiamoebic drug; although some authors consider that a value like this is within the accepted range [49,50], other authors consider it appropriate to re-evaluate the toxicity using other biosystem(s) when the SI is between 1 and 10 in order to determine whether or not the sample is a candidate for further study [51].

The essential oil of *P. amboinicus* showed a weak or moderate inhibitory effect against epimastigotes with an IC_50_ of 210.8 ± 28.2 µg/mL and a low SI (4.16); therefore, it might also be necessary to reassess its toxicity using other methods to define its therapeutic potential against *T. cruzi* epimastigotes. In contrast, the essential oil exhibited a strong effect against *T. cruzi*, trypomastigotes with an LC_50_ of 61.66 ± 8.42 µg/mL, which is very close to that of the reference drug benznidazole (98.83 µM; 26 µg/mL) [52]; furthermore, the SI obtained was adequate given its low toxicity in mammalian cells, which could be useful for its application during the acute phase of the disease to interfere with its progression and the transmission of the parasite, since in this phase blood trypomastigotes infect other host cells and also the triatomine bugs that feed on this blood [53].

As we mentioned previously, other authors have reported that several of the most abundant molecules in the essential oil of *P. amboinicus* have effects on mitochondrial protozoa such as trypanosomatids and plasmodium, which could be related to our results, where the essential oil has a greater effect against *T. cruzi*. However, the susceptibility of *T. cruzi* and *E. histolytica* to the essential oil is not only determined by the individual metabolites present in it, but also by their abundance, their pharmacological interactions (as synergism or antagonism) and their targets, which could be different since *E. histolytica* and *T. cruzi* are two organisms with characteristics inherent to their taxonomic groups. Minor metabolites present in our essential oil such as limonene, thymol, γ-terpinene, p-cymene, and β-caryophyllene, among others, could also be contributing to the observed effect.

It would also be very interesting to also evaluate the effect of the essential oil in experimental models of amebiasis and trypanosomiasis, taking as a precedent a previous report about its antimalarial activity in mice infected with *P. berguei*, in which the oil has a prophylactic and curative effects, decreasing parasitemia by up to 58% and 65%, respectively [12].

In conclusion, this first study on the essential oil from *P. amboinicus* cultivated in Mexico City evidenced that the major compound is camphor, followed by alloaromadendrene oxide, caryophyllene oxide, aromadendrene oxide, and carvacrol. The essential oil possesses antiamebic activity and antitrypanosomiasis activity against trypomastigotes and epimastigotes, being more effective against *T. cruzi* trypomastigotes.

Even though some mechanisms of action of molecules present in the essential oil of *P. amboinicus* have already been described, future research will allow us to characterize the molecular effects that the essential oil of *P. amboinicus* cultivated in Mexico has on the protozoan parasites *E. histolytica* and *T. cruzi.* It would also be important to compare the activity of the essential oil, with respect to the activity of its metabolites (alone or mixed) and its effect in combination with the reference drugs.

## Figures and Tables

**Table 1:** Chemical composition of the essential oil from *P. amboinicus* grown in Mexico. The number of the peak, the retention time (min), the name of the identified compound, the molecular formula, the compound class and the abundance of each molecule (%) are shown; values are given as mean ± SD (n = 3). Data of the five most abundant compounds are in bold.

Peak Num.	Retention Time (min)	Identified Compound	Molecular Formula	Class	Abundance (%) ± SD
1	8.900	α-Pinene	C_10_H_16_	Monoterpene hydrocarbon	0.23 ± 0.07
2	9.415	Camphene	C_10_H_16_	Monoterpene hydrocarbon	1.07 ± 0.20
3	10.548	β-Pinene	C_10_H_16_	Monoterpene hydrocarbon	0.41 ± 0.13
4	11.023	1-Octen-3-ol	C_8_H_16_O	Secondary alcohol	0.42 ± 0.21
5	11.304	β-Myrcene	C_10_H_16_	Monoterpene hydrocarbon	0.12 ± 0.06
6	12.191	α-Terpinene	C_10_H_16_	Monoterpene hydrocarbon	2.27 ± 1.19
7	12.505	p-Cymene	C_10_H_14_	Monoterpene hydrocarbon	4.58 ± 1.27
8	12.637	D-Limonene	C_10_H_16_	Monoterpene hydrocarbon	0.60 ± 0.00
9	12.729	Eucalyptol	C_10_H_18_O	Oxygenated monoterpene	3.05 ± 2.65
10	13.461	3-Carene	C_10_H_16_	Monoterpene hydrocarbon	0.08 ± 0.00
11	13.776	γ-Terpinene	C_10_H_16_	Monoterpene hydrocarbon	0.27 ± 0.06
12	14.823	Terpinolene	C_10_H_16_	Monoterpene hydrocarbon	0.37 ± 0.43
13	14.823	β-Linalool	C_10_H_18_O	Oxygenated monoterpene	0.18 ± 0.04
14	16.064	trans-p-Mentha-2,8-dienol	C_10_H_16_O	Oxygenated monoterpene/Monoterpene alcohol	0.11 ± 0.02
15	16.379	Cyclohexanone, 2-pentyl-	C_11_H_20_O	Monocyclic alicyclic ketone	0.23 ± 0.00
**16**	**16.808**	**Camphor**	**C_10_H_16_O**	**Oxygenated monoterpene**	**30.64 ± 1.95**
17	17.295	L-α-Terpineol	C_10_H_18_O	Oxygenated monoterpene/Monoterpene alcohol	0.56 ± 0.61
18	17.764	endo-Borneol	C_10_H_18_O	Oxygenated monoterpene/Monoterpene alcohol	0.96 ± 0.05
19	17.878	Terpinen-4-ol	C_10_H_18_O	Oxygenated monoterpene/Monoterpene alcohol	0.85 ± 0.18
20	18.210	D-Verbenone	C_10_H_14_O	Oxygenated monoterpene/Monoterpenoid ketone	0.15 ± 0.00
21	18.319	α-Terpineol	C_10_H_18_O	Oxygenated monoterpene/Monoterpene alcohol	1.67 ± 2.29
22	18.502	2-Pinen-10-ol	C_10_H_16_O	Oxygenated monoterpene/Monoterpene alcohol	0.23 ± 0.07
23	19.423	5-Isopropenyl-2-methyl-7-oxabicyclo[4.1.0]heptan-2-ol	C_10_H_16_O_2_	Oxygenated monoterpene	0.31 ± 0.01
24	19.789	2-Carene	C_10_H_16_	Monoterpene hydrocarbon	1.62 ± 1.10
25	20.379	7-Oxabicyclo[4.1.0]heptan-2-one, 3-methyl-6-(1-methylethyl)-	C_10_H_16_O_2_	Oxygenated monoterpenoid	0.48 ± 0.34
26	20.854	2-Isopropyl-5-methyl-6-oxabicyclo[3.1.0]hexane-1-carboxaldehyde	C_10_H_16_O_2_	Oxygenated monoterpenoid/ monoterpene aldehyde	0.14 ± 0.03
27	21.203	α-Terpenyl acetate	C_10_H_18_O_2_	Oxygenated monoterpene	0.09 ± 0.05
28	21.454	Ascaridole	C_10_H_16_O_2_	Oxygenated monoterpene	0.18 ± 0.00
29	21.678	Thymol	C_10_H_14_O	Oxygenated monoterpene	1.38 ± 1.96
**30**	**21.901**	**Carvacrol**	**C_10_H_14_O**	**Oxygenated monoterpene**	**5.80 ± 2.25**
31	23.520	Ascaridole epoxide	C_10_H_16_O_3_	Oxygenated monoterpene	0.27 ± 0.00
32	23.869	α-Copaene	C_15_H_24_	Sesquiterpene hydrocarbon	0.13 ± 0.02
33	24.121	1-Hydroxy-1,7-dimethyl-4-isopropyl-2,7-cyclodecadiene	C_15_H_26_O	Oxygenated monoterpene/Sesquiterpenoid alcohol	0.11 ± 0.00
34	24.321	(-)-β-Elemene	C_15_H_24_	Sesquiterpene hydrocarbon	0.08 ± 0.04
35	25.082	Caryophyllene/β-Caryophyllene	C_15_H_24_	Sesquiterpene hydrocarbon	0.82 ± 0.64
36	26.089	Humulene	C_15_H_24_	Sesquiterpene hydrocarbon	0.33 ± 0.31
37	27.005	β-Cubebene	C_15_H_24_	Sesquiterpene hydrocarbon	0.35 ± 0.24
38	27.182	β-Eudesmene	C_15_H_24_	Sesquiterpene hydrocarbon	0.46 ± 0.18
39	27.611	cubebol	C_15_H_26_O	Oxygenated sesquiterpene	0.59 ± 0.15
40	28.258	β-Selinene	C_15_H_24_	Sesquiterpene hydrocarbons	0.32 ± 0.11
41	28.624	δ-Cadinene, (+)-	C_15_H_24_	Oxygenated monoterpene/Sesquiterpene alcohol	0.83 ± 0.05
42	30.896	(-)-Isolongifolol, acetate	C_17_H_28_O_2_	Sesquiterpene hydrocarbon	1.42 ± 0.00
**43**	**31.205**	**Caryophyllene oxide**	**C_15_H_24_O**	**Oxygenated sesquiterpene/Sesquiterpenoid ester**	**6.61 ± 1.23**
44	31.611	Isoaromadendrene epoxide	C_15_H_24_O	Oxygenated sesquiterpene/Sesquiterpenoid ester	1.58 ± 1.87
45	32.137	Viridiflorol	C_15_H_26_O	Oxygenated sesquiterpene	1.24 ± 0.51
46	32.418	Globulol	C_15_H_26_O	Oxygenated sesquiterpene	1.89 ± 1.12
47	33.219	β-Eudesmol	C_15_H_24_O	Oxygenated sesquiterpene	1.62 ± 0.43
**48**	**34.209**	**Alloaromadendrene oxide-(1)**	**C** **_15_** **H** **_24_** **O**	**Oxygenated sesquiterpene**	**14.43 ± 1.88**
**49**	**34.775**	**Aromadendrene oxide-(2)**	**C** **_15_** **H** **_24_** **O**	**Oxygenated sesquiterpene**	**6.47 ± 0.16**

**Table 2:** Antiparasitic activity, cytotoxicity and selectivity index of the essential oil of *P. amboinicus.* The data were obtained at 24 or 48 h with a 95% confidence interval. n = 3.

Parasite	IC_50_ or LC_50_ (μg/mL)	CC_50_ Vero Cells (μg/mL)	Selectivity Index (SI)
*E. histolytica* (trophozoites)	121.7 (48 h)	364.4 (48 h)	2.99
*T. cruzi* (trypomastigotes)	61.66 (24 h)	876.4 (24 h)	14.21
*T. cruzi* (epimastigotes)	210.8 (24 h)	876.4 (24 h)	4.16

## Data Availability

The original contributions presented in this study are included in the article. Further inquiries can be directed to the corresponding author.
